# Supplementary material for: Over-expression and increased copy numbers of a cytochrome P450 and two UDP-glucuronosyltransferase genes in macrocyclic lactone resistant Psoroptes ovis of cattle
Source: PLoS Pathog. 2025 Jul 29;21(7):e1012963. doi: 10.1371/journal.ppat.1012963 (PMC12364353; doi:10.1371/journal.ppat.1012963)
Supplement: S1 Methods — (DOCX) [file ppat.1012963.s001.docx]

**Supplementary Methods S1**

**Supplementary Methods for Section 2.1**

**Section 2.1.3. DNA extraction**

The temperature of a pre-chilled mortar (overnight at -80°C) was further lowered with liquid nitrogen. When most nitrogen had evaporated, 25 previously snap-frozen mites were submerged in a thin layer of liquid nitrogen. ATL-buffer (180 µl per 25 mites, Qiagen, DE) was added when all the liquid nitrogen had evaporated. The mites were crushed with a pestle to a fine powder and transferred to a new 1.5 ml eppendorf tube. To each sample, 20 µl proteinase K (Qiagen, DE) was added and the solution was incubated overnight in a shaking heat block at 56°C at 300 RPM. Five hundred µl phenol:chloroform:IAA (25:24:1; Sigma-Aldrich, USA) was added. The mixture was briefly vortexed and centrifuged for 10 minutes at 10,000 g (4°C). The supernatant (≈ 400 µl) was transferred to a new 1.5 ml eppendorf tube and 40 µl (=1/10) sodium acetate (3M; pH 5.2; Sigma-Aldrich, USA) and 400 µl (=1/1) isopropanol (Biosolve, NL) were added. After centrifuging for 10 minutes at 16,000 g (4°C), the supernatant was discarded.

Six hundred µl 80% ethanol (Sigma-Aldrich, USA) was added to the pellet and centrifuged for 5 minutes at 10,000 g (4°C). After discarding the supernatant, this washing step was repeated once. Finally, the pellet was dried at room temperature (~20°C) until the ethanol had evaporated (~5 to 15 minutes) and subsequently dissolved in 25 µl nuclease-free water (Ambion, USA). The DNA concentration was measured with a NanoDrop™ 2000 spectrophotometer (Thermo Fisher Scientific, USA).

**Section 2.1.3. High fidelity PCR and purification**

Fragments (179 bp long) from the two different GluCls in *P. ovis* were amplified according to the Phusion High-Fidelity polymerase chain reaction protocol (Thermo Fisher, USA). The forward primer for GluCl-44 was 5’ AGCCGCCTGTCAGTTATACG 3’ and the reverse primer 5’ GCAATCCATAAGCAGCCAAT 3’. For GluCl-280, the forward primer was 5’ ACATCCATCAGTGGCATCAA 3’ and the reverse primer 5’ GGCTGAACCATGTTCGAGAT 3’ (detailed in Supplementary Table S2. The thermocycling parameters were 98°C for 30 s, followed by 30 cycles of 98°C for 5 s, 62°C for 10 s, 72°C for 15 s, and a final extension of 72°C for 5 min. PCR products were purified from agarose gels with the Zymoclean Gel DNA Recovery kit (Zymo Research, USA), following the manufacturers’ protocol.

**Section 2.1.3. PCR amplification of the GluCl-44 and GluCl-280 fragments**

These primers were adjusted for Illumina deep sequencing and for both genes, four forward and four reverse primers were mixed in equal concentrations (primer design, Supplementary Table S2). The Illumina adaptor oligonucleotide sequences were obtained from the Illumina Adapter Sequences document (Oligonucleotide sequences 2020 Illumina, Inc.) and added to allow later annealing of sequencing primers. For each of the primers, 0-3 random nucleotides were inserted between the locus specific primer sequence and the Illumina Adapter sequence. These nucleotides prevent oversaturation of the MiSeq sequencing channels by increasing the diversity of generated amplicons. PCR conditions as follows: 5 μL KAPA HiFi HotStart Fidelity Buffer (5X) (KAPA Biosystems, USA), 0.75 μL Forward Primer Mix (10 μM), 0.75 μL Reverse Primer Mix (10 μM), 0.5 μL dNTPs (10 mM), 0.5 μL KAPA HiFi HotStart Polymerase (0.5 U), 16.4 μL molecular-grade H_2_O, 0.1 μL BSA (20mg/mL) and 1μL of 1:10 dilution of gel-purified PCR product. The thermocycling parameters were 95°C for 3 min, followed by 30 cycles of 98°C for 10s, 65°C for 15s, 72°C for 15s, and a final extension of 72°C for 2 min. PCR products were purified with AMPure XP Magnetic Beads (1X) (Beckman Coulter, Inc., USA) following the manufacturer’s recommended protocol. All samples were eluted in 32.5 μL of molecular-grade H_2_O.

By limited cycle PCR amplification, both Illumina barcode indices and P5/P7 sequencing regions were added to the GluCl-44 and GluCl-280 amplicons. Unique forward/reverse combinations of primers of the Nextera XT Index Kit v2 set (Oligonucleotide sequences 2020 Illumina, Inc.) were made, enabling individual sample barcoding. The following PCR conditions were used: 5 μL KAPA HiFi HotStart Fidelity Buffer (5X) (KAPA Biosystems, USA), 2.5 μL Forward Primer (S502-S511) (5 μM), 2.5 μL Reverse Primer (N716-N719) (5 μM), 0.75 μL dNTPs (10 mM), 0.5 μL KAPA HiFi HotStart Polymerase (0.5 U), 8.75 μL molecular-grade H_2_O and 5μL of first-round clean PCR product as template. The thermocycling parameters were 98°C for 45s, followed by seven cycles of 98°C for 20s, 63°C for 20s, 72°C for 2 min. Amplicons were purified with AMPure XP Magnetic Beads (1X) (Beckman Coulter, Inc., USA) as previously described.

The concentration of the second-round clean PCR product was measured using the Implen (DE) NanoPhotometer NP80 and 50ng of each sample was pooled to produce a master sequencing library. The final concentration of this pooled library was assessed with the KAPA qPCR Library Quantification Kit (KAPA Biosystems, USA), following the manufacturer’s recommended protocol. The prepared pooled library was run on an Illumina MiSeq Desktop Sequencer using a 2x250 v2 reagent kit (MiSeq Reagent Kits v2, MS-103-2003) at a concentration of 15 pM with the addition of 20% PhiX Control v3 (Illumina, FC-110-3001). The MiSeq was set to generate only FASTQ files with no post-run analysis. Based on their supplied index combinations, samples were automatically demultiplexed by the MiSeq. All protocols were carried out per Illumina’s standard MiSeq operating protocol (Illumina, Inc., USA).

**Supplementary Methods for Section 2.2**

**Section 2.2. Long-Read Whole Genome Sequencing (WGS) Library Preparation**

Genomic DNA from *P. ovis* isolated from sheep (UK) and from cattle (Belgium) was extracted from adult female *P. ovis* mites and quality-controlled for long-read sequencing, considering purity, quantity and integrity. The sequencing libraries were prepared using the ligation 1D sequencing kit 9 or upgraded kit 10 chemistry (SQK-LSK109 or SQK-LSK110, respectively) from Oxford Nanopore Technologies (ONT). In brief, 3.0 μg of the gDNA underwent end-repair and adenylation using the NEBNext UltraII End Repair/dA-Tailing Module (NEB), followed by ligation of sequencing adaptors. The ligation product was purified using 0.4X AMPure XP Beads and eluted in Elution Buffer (ONT). The WGS long-read sequencing run was performed on a GridIon Mk1 (ONT) using a flow cell R9.4.1 (FLO-MIN106) compatible with sequencing ligation kit 9 and 10. The sequencing data was collected for 72 hours. The quality parameters of the sequencing runs were monitored in real time using the MinKNOW platform version 4.2.5, and basecalling was performed using Guppy version 4.3.4.

**Section 2.2. Short-Read Whole Genome Sequencing (WGS) Library Preparation**

The short-insert paired-end library for whole genome sequencing was prepared using the PCR-free protocol and the KAPA HyperPrep kit (Roche). After end-repair and adenylation, Illumina platform-compatible adaptors with unique dual indexes and unique molecular identifiers (Integrated DNA Technologies) were ligated. The sequencing libraries were quality controlled on an Agilent 2100 Bioanalyzer using the DNA 7500 assay (Agilent) to assess size and quantified using the Kapa Library Quantification Kit for Illumina platforms (Roche).

**Section 2.2. 10X Genomics library preparation and short read sequencing**

The linked read libraries from each isolate of gDNA were prepared using the Chromium Controller instrument (10x Genomics) and Genome Reagent Kits v2 (10x Genomics) following the manufacturer’s protocol. Briefly, 10 ng of high molecular weight genomic DNA (HMW gDNA) was portioned in GEM reactions, including a unique barcode (Gemcode), after being loaded onto a chromium controller chip. The droplets were then recovered, isothermally incubated, and fractured, and the intermediate DNA library was then purified and size-selected using Silane and Solid Phase Reverse Immobilisation (SPRI) beads. Illumina-compatible paired-end sequencing libraries were prepared following 10X Genomics recommendations and validated on an Agilent 2100 BioAnalyzer with the DNA 7500 assay (Agilent). The 10X Genomics and WGS libraries were sequenced on Illumina NovaSeq 6000 with a read length of 2x151bp, following the manufacturer’s protocol for dual indexing. Image analysis, base calling, and quality scoring of the run were processed using the manufacturer's software, Real Time Analysis (RTA 3.4.4). Read sequences and assemblies for the sheep-derived and cattle-derived *P. ovis* assemblies are available under ENA project PRJEB84953 in sub-projects PRJEB82899 and PRJEB82900 respectively).

**Supplementary Methods for Section 2.3**

**Section 2.3.1. RNA extraction and quality control**

A glass homogenizer (7 ml, Kontes, USA) was soaked in 0.1% DEPC for 12 hours at room temperature (~20 °C). A total of 150 frozen adult female mites were put into the glass homogenizer and 1 ml TRIZol (Ambion, USA) was added before homogenisation. Samples were homogenised until all macroscopically visible particles in the solution were gone. One ml of the homogenised mixture was transferred into a 2 ml tube and incubated for 5 minutes at room temperature, (~20 °C). The samples were then centrifuged at 12,000 g for 10 minutes in a pre-chilled rotor (4°C) and the supernatant transferred to a new 2 ml tube. Two hundred µl chloroform (Sigma-Aldrich, USA) was added to each tube and the tubes were inverted end-on-end 10 times. The samples were then centrifuged at 10,000 g (4°C) for 15 minutes and the top aqueous phase carefully transferred to fresh 2 ml tubes. Five hundred µl isopropanol (2-propanol; Biosolve, NL) was added to each aqueous phase sample and the tubes were inverted end on end 10 times. The samples were then centrifuged at 12,000 g (4°C) for 15 minutes and the supernatant discarded. One ml of 75% ethanol (Sigma-Aldrich, USA) was added to each pellet and then mixed by vortexing. The samples were subsequently centrifuged at 7,500 g (4°C) for 5 minutes. The ethanol was decanted, and the pellets were air dried for 1-2 minutes at room temperature (~20 °C). The RNA-pellet was resuspended in 50 µl RNase free water (Ambion, USA) and stored at -80°C. RNA quantity was determined with the NanoDrop 2000 (Thermo Fisher Scientific, USA). RNA quality was determined with the Experion™ Automated Electrophoresis Station (Bio-Rad, USA). RNA concentrations after extraction ranged from 102.6 ng/µl to 383.0 ng/µl and the RNA integrity number (RIN) was at least 8.3.

**Section 2.3.2 Quantification and differential expression of RNASeq data**

All 9 replicates were diluted to a final concentration of 40 ng/µl RNA with nuclease free water (Ambion, USA). From the RNA samples, Illumina libraries were constructed with the TruSeq Stranded mRNA Library Preparation Kit (Illumina, USA), and the resulting libraries were sequenced on a NovaSeq 6000 to generate strand-specific paired reads of 2 × 100 bp. Library construction and sequencing was performed at Fasteris, Geneva, Switzerland and raw reads are available under project PRJEB82994 in the ENA. Post-sequencing, read quality of raw FASTQ files was checked with FastQC v0.11.9.

**Section 2.3.3. Validation of RNA-seq data by Real Time qPCR**

qPCR reactions were run on a StepOnePlus real-time PCR system (Applied Biosystems, USA). cDNA was diluted 1:8 in nuclease free water and Fast SYBR Green Master Mix Real-Time PCR Master Mix was used following the recommended protocol. Standard curves for all genes were based on a pooled sample from SUS1, SUS2 and SUS 3 in 1:2, 1:4, 1:8, 1:16 and 1:32 dilution. All samples were run in duplicate. PCR conditions were as follows: 1 cycle of 95°C for 20 s, 40 cycles of 3 s at 95°C and 30 s at 60°C (optimal annealing temperature for all examined genes) and 1 cycle of 15 s at 95°C, 60 s at 60°C and 15 s at 95°C. Relative quantities of gene expression were calculated using the delta Ct method to determine the fold differences in gene transcription levels of the genes in the different mite populations. Housekeeping genes were used for normalisation with Genorm [1].

1. Vandesompele J, De Preter K, Pattyn F, Poppe B, Van Roy N, De Paepe A, et al. Accurate normalization of real-time quantitative RT-PCR data by geometric averaging of multiple internal control genes. Genome Biol. 2002;3: 1–12.
