# Supplementary material for: Over-expression and increased copy numbers of a cytochrome P450 and two UDP-glucuronosyltransferase genes in macrocyclic lactone resistant Psoroptes ovis of cattle
Source: PLoS Pathog. 2025 Jul 29;21(7):e1012963. doi: 10.1371/journal.ppat.1012963 (PMC12364353; doi:10.1371/journal.ppat.1012963)
Supplement: S2 Methods — (DOCX) [file ppat.1012963.s002.docx]

**Supplementary Methods S2**

**Genome assembly methodology, evaluation and results for sheep- and cattle-derived *P. ovis* assemblies.**

Jessica Gomez-Garrido^1,2^, Fernando Cruz^1,2^, Marta Gut^1,2^, Tyler Alioto^1,2^

1. Centro Nacional de Análisis Genómico (CNAG), Baldiri Reixac 4, 08028 Barcelona, Spain.

2. Universitat de Barcelona (UB), Barcelona, Spain.

## Methods

*Genome assembly*

For the sheep-derived sample of *P. ovis* (qqPsoOvis1**)**, the genome was assembled using a combination of ONT long reads and 10X Genomics linked reads sequenced with Illumina paired-end technology.

Prior to assembly, the 10X Genomics Illumina reads were processed with Longranger basic to remove the barcode and adapter information and put the barcode information in the FASTQ header. A k-mer database (k=18) was subsequently built with Jellyfish [(1)](https://sciwheel.com/work/citation?ids=1009601&pre=&suf=&sa=0&dbf=0) and used as input to Genomescope2 [(2)](https://sciwheel.com/work/citation?ids=10208900&pre=&suf=&sa=0&dbf=0) to estimate haploid genome size, heterozygosity and repeat content. No appreciable heterozygosity was recognized, so ploidy was set to 1. The genome size was estimated to be 80.7 Mb and kcov=115.

The quality parameters of the sequencing run for sheep and cattle-derived ONT data were monitored in real time using the MinKNOW platform versions 4.2.5 and 5.4.3, respectively. The base-calling was performed using Guppy versions 4.3.4 in high-accuracy mode and 6.4.6 in super-accuracy mode, respectively for sheep and cattle-derived ONT dataset. The resulting 21.5 Gb of ONT data were filtered with Filtlong (<https://github.com/rrwick/Filtlong>; --minlen 5000 --target_bases 4000000000) to remove short and low-quality reads, keeping only 4 Gb of the longest highest quality long read data (read length N50 = 32 kb, median read quality = 9.7).

The filtered ONT data was assembled with Flye v2.8.3 [(3)](https://sciwheel.com/work/citation?ids=6744810&pre=&suf=&sa=0&dbf=0) with two iterations of the internal Flye polisher (option -i). To improve the base accuracy of the assembly, it was polished three times with HyPo [(4)](https://sciwheel.com/work/citation?ids=9175099&pre=&suf=&sa=0&dbf=0) using both Illumina and ONT data. The result was 13 contigs greater than 2 Mb in length and five contigs less than 45 kb. To remove potential contamination, we examined read coverage (ONT reads were aligned back to the assembly with minimap2) as well as taxonomic identification by searching against the NCBI nt database using megablast. Only those contigs with hits to mite sequences and with the expected read coverage were retained. One large contig (contig_1) of 5.5 Mb in length corresponded to *Serratia ureilytica* and was removed from the assembly. This likely corresponds to an endosymbiont. One of the small contigs (contig_21, 16878 bp) was retained as it has hits to the dust mite *Dermatophagoides pteronyssinus.* To confirm the list of contaminants we also ran the Blobtoolkit pipeline [(5)](https://sciwheel.com/work/citation?ids=8266351&pre=&suf=&sa=0&dbf=0) using the NCBI nt database and several BUSCO odb10 databases (metazoa, eukaryota, fungi and bacteria) (**Figure 1. SheepDerivedBlobtools**). The decontaminated assembly was scaffolded with 10X Linked-Reads using the Faircloth’s Lab pipeline ([http://protocols.faircloth-lab.org/en/latest/protocols-computer/assembly/assembly-scaffolding-with-arks-and-links.html#](http://protocols.faircloth-lab.org/en/latest/protocols-computer/assembly/assembly-scaffolding-with-arks-and-links.html)). This pipeline includes Tigmint v1.1.2 [(6)](https://sciwheel.com/work/citation?ids=5956327&pre=&suf=&sa=0&dbf=0), ARKS v1.0.3 [(7)](https://sciwheel.com/work/citation?ids=8893228&pre=&suf=&sa=0&dbf=0), and LINKS v1.8.5 [(8)](https://sciwheel.com/work/citation?ids=722355&pre=&suf=&sa=0&dbf=0). Two joins were made, resulting in ten large sequences (eight contigs and two scaffolds) and one small contig accounting for 62,663,122 bp with an N50 of 7.33 Mb (Table XX).

**
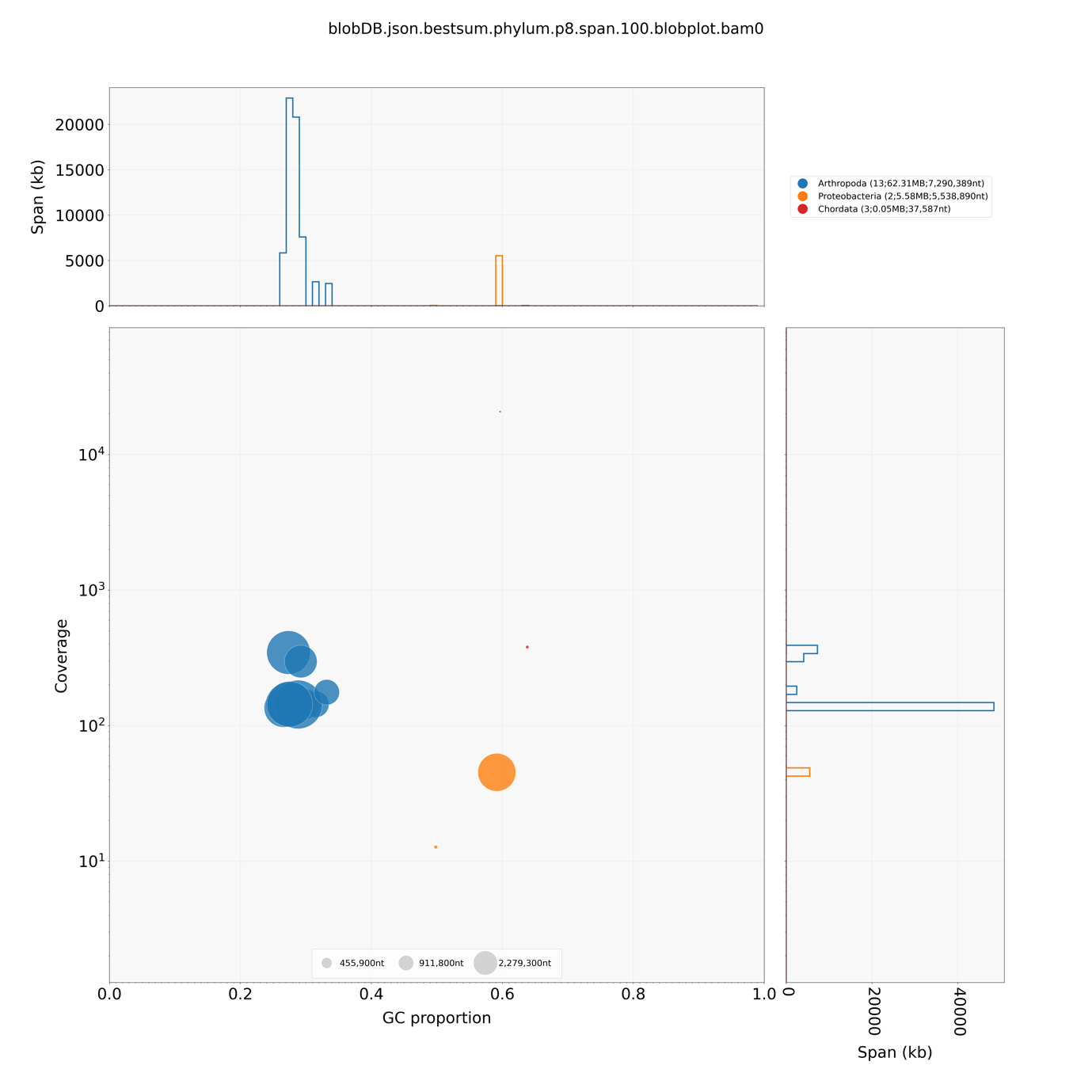
**

**Figure 1. SheepDerivedBlobtools.** Scaffolds are plotted according to GC content (x-axis), sequencing coverage (y-axis), length (circle size) and taxa by best sum-order (by color, see legend).

For the cattle-derived sample of *P. ovis* (qqPsoOvis2**),** the genome was assembled using the CLAWS v2.1 workflow [(9)](https://sciwheel.com/work/citation?ids=16555987&pre=&suf=&sa=0&dbf=0) combining ONT long reads and 10X linked reads.

Prior to assembly, the 10X Genomics Illumina reads were processed with Longranger basic to remove the barcode and adapter information and put the barcode information in the FASTQ header. A k-mer database was subsequently built with Meryl (<https://github.com/marbl/meryl>). The k-mer histogram (k=18) generated by Meryl was used as input to Genomescope2 [(2)](https://sciwheel.com/work/citation?ids=10208900&pre=&suf=&sa=0&dbf=0) to estimate haploid genome size, heterozygosity and repeat content. The k-mer depth (kcov = 28) did not match the sequencing depth and the estimated genome size of 277 Mb was much larger than the assembly span obtained with the sheep-derived sample, suggesting large amounts of contamination. We decided to proceed with assembly as usual and decontaminate after assembly.

The 12.2 Gb of ONT data were filtered with Filtlong (<https://github.com/rrwick/Filtlong>; --minlen 1000 --min_mean_q 80 --target_bases 3600000000) to remove short and low-quality reads, keeping only 60x of the longest highest-quality long read data (read length N50 = 13 kb, median read quality = 14.3). Despite higher quality, the read lengths obtained from this sample were much shorter than those of the sheep-derived sample. A target of 100x was also tried but results were worse.

The filtered ONT data was assembled with both NextDenovo [(10)](https://sciwheel.com/work/citation?ids=15387919&pre=&suf=&sa=0&dbf=0) and Flye v2.9.1 [(3)](https://sciwheel.com/work/citation?ids=6744810&pre=&suf=&sa=0&dbf=0) with the –scaffold option and 2 iterations of internal polishing. The Flye assembly was more complete (85% BUSCO completeness vs. 71% for Nextdenovo, see Supl. Table ASS_NEW), so we continued with the Flye assembly. To improve the base accuracy of the assembly, it was polished with HyPo [(4)](https://sciwheel.com/work/citation?ids=9175099&pre=&suf=&sa=0&dbf=0) using both Illumina and ONT data. Finally, the polished assembly was purged with purge_dups [(11)](https://sciwheel.com/work/citation?ids=8159585&pre=&suf=&sa=0&dbf=0) to remove alternate haplotypes and other artificially duplicated repetitive regions, reducing the number of scaffolds from 1650 to 1412 with an N50 of 335 kb. As this assembly was of low contiguity, the assembly was then aligned to the sheep-derived assembly and reference-based scaffolding was carried out using RagTag [(12)](https://sciwheel.com/work/citation?ids=14687793&pre=&suf=&sa=0&dbf=0).

To identify and remove potential contamination, Blobtoolkit [(5)](https://sciwheel.com/work/citation?ids=8266351&pre=&suf=&sa=0&dbf=0) pipeline was run, using the NCBI nt database and several BUSCO odb10 databases (metazoa, eukaryota, fungi and bacteria). Contaminants were defined as scaffolds with either GC content > 0.35, zero coverage, or with matches to Bacillota. Ten superscaffolds and 14 unplaced scaffolds, with a total span of 62,549,845 bp, were retained after this step (**Figure 2. Blobplots**).

| A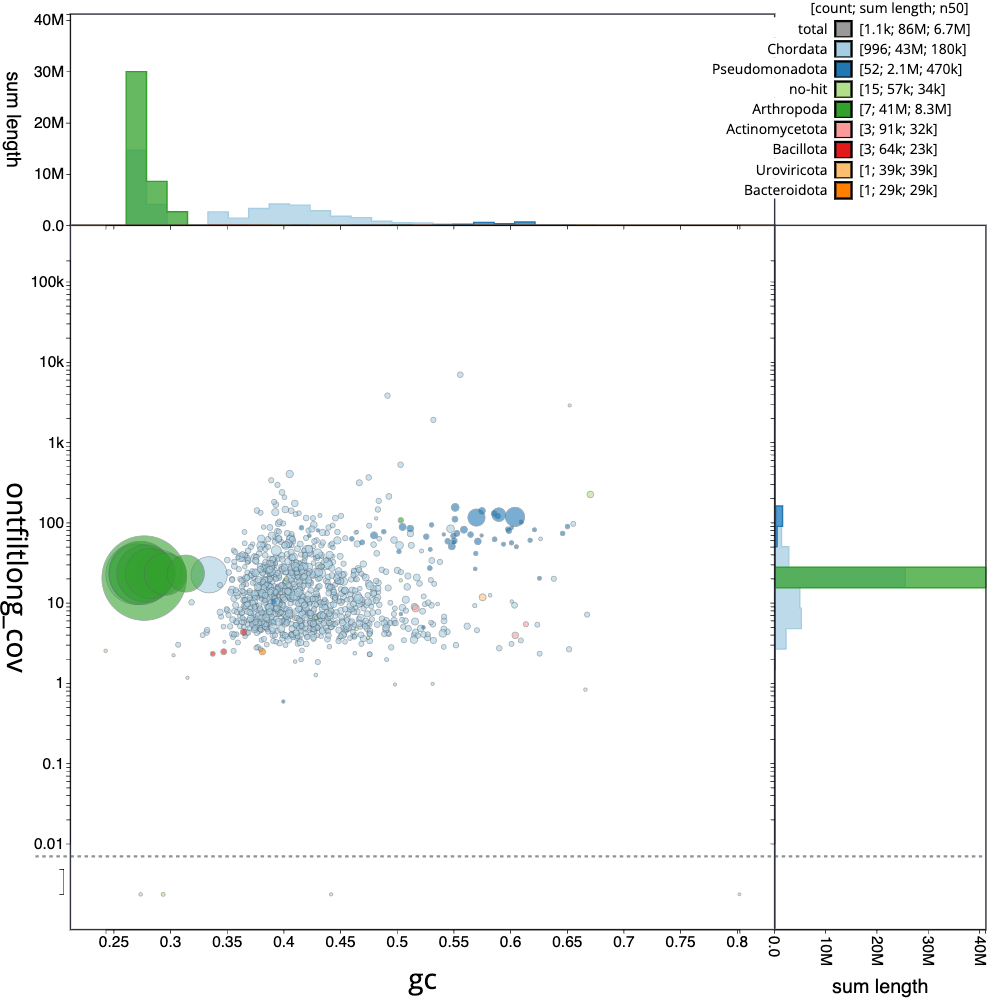 |
| --- |
| B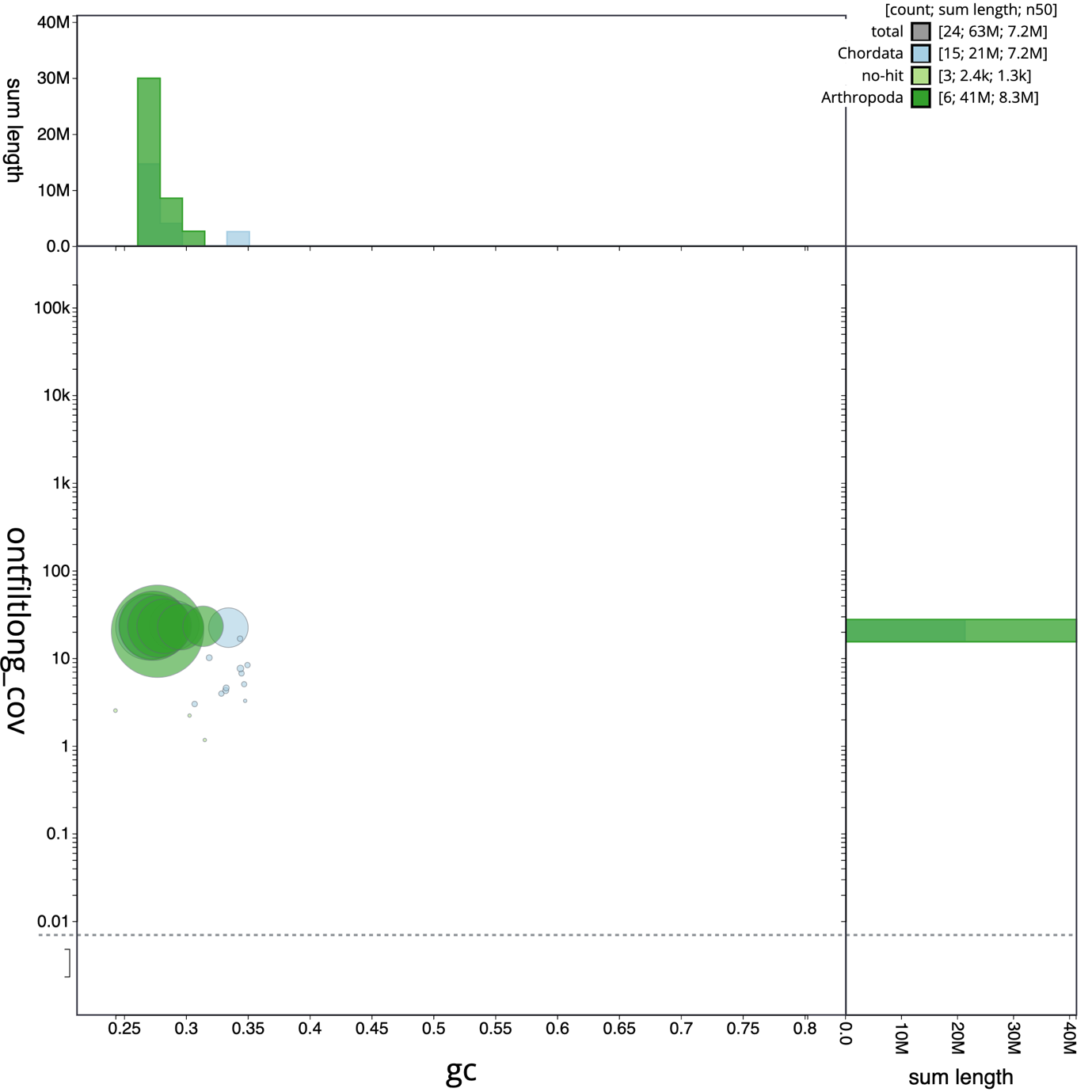 |

**Figure 2. Blobplots.** Scaffolds are plotted according to GC content (x-axis), sequencing coverage (y-axis), length (circle size) and taxa by best sum-order (by color, see legend) before (a) and after (b) filtering.

To estimate the accuracy and completeness of the genome assemblies, BUSCO [(13)](https://sciwheel.com/work/citation?ids=11841494&pre=&suf=&sa=0&dbf=0) and Merqury [(14)](https://sciwheel.com/work/citation?ids=9829443&pre=&suf=&sa=0&dbf=0) (Figure 3 and 4) were run. Snailplots and blobplots were produced with Blobtoolkit.

## Results and Discussion

*Genome assembly*

The chosen Flye assembly span (62.7 Mb) and number of large scaffolds (10) are close to the expected size and number of chromosomes (another less contiguous assembly, GCA_002943765.1, also has a span of 63 Mb and the GoaT estimate of haploid number is nine based on ancestral genomes). Genome assembly statistics are reported in **Table 1.**

**Table 1. Genome metrics**

|  | Sheep-derived (qqPsoOvis1.1) | Cattle-derived (qqPsoOvis2.1) |
| --- | --- | --- |
| Span | 62,663,122 | 62,549,845 |
| Contig N50 | 7,334,676 | 491,361 |
| Scaffold N50 | 7,334,676 | 7,163,830 |
| # contigs | 13 | 260 |
| # scaffolds | 11 | 24 |
| Merqury Consensus quality | 43.3 | 42.7 |
| BUSCO (arthropoda_odb10) | C:89.5%[S:88.9%,D:0.6%],  F:1.0%,M:9.5%,n:1013 (v4.0.6) | C:85.9%[S:85.5%,D:0.4%],  F:4.6%,M:9.5%,n:1013 (v5.4.0) |

**Comparison of genome assemblies**

Table 2 shows statistics computed for all the assemblies obtained during the assembly process. Not only was Busco completeness was higher for the Flye assemblies, but also contig and scaffold N50s. Additionally, the length of the Nextdenovo assembly seems to suggest that not all the genome is present in the assembly.

**Table 2**. Genome Assembly metrics for all intermediate assemblies produced by CLAWS v2.1 for the cattle-derived strain

| **assembly** | **cN50** | **cL50** | **sN50** | **sL50** | **total_len** | **total_seq** | **BUSCOv5** | **Merqury**  **QV** | **Merqury**  **completenes** | **Merqury**  **False dups** |
| --- | --- | --- | --- | --- | --- | --- | --- | --- | --- | --- |
| Flye | 313.970 | 75 | 313.970 | 75 | 94.599.076 | 1.650 | C:84.9%[S:83.5%,D:1.4%],  F:4.9%,M:10.2%,n:1013 | 27,2003 | 68,0403 | 0,5517 |
| Flye + hypo | 314.394 | 75 | 314.394 | 75 | 94.530.401 | 1.650 | C:86.3%[S:85.0%,D:1.3%],  F:4.3%,M:9.4%,n:1013 | 30,0328 | 69,6647 | 0,4280 |
| Flye + hypo + purged | 335.021 | 68 | 335.021 | 68 | 86.192.300 | 1.314 | C:86.2%[S:85.2%,D:1.0%],  F:4.4%,M:9.4%,n:1013 | 30,3370 | 38,0145 | 0,3808 |
| Flye + hypo + purged +  10X scaffolding | 318.479 | 69 | 342.550 | 65 | 86.192.410 | 2.884 | C:86.2%[S:85.3%,D:0.9%],  F:4.4%,M:9.4%,n:1013 | 30,3393 | 38,0107 | 0,3808 |
| Flye + hypo + purged +  10X scaffolding + ragtag | 335.021 | 68 | 6.692.840 | 5 | 86.215.900 | 1.078 | 86.0%[S:85.3%,D:0.7%],  F:4.5%,M:9.5%,n:1013 | 30,3370 | 38,0145 | 0,3808 |
| Flye + hypo + purged +  10X scaffolding + ragtag + decont | 491.361 | 40 | 7.163.830 | 4 | 62.549.845 | 24 | C:85.9%[S:85.5%,D:0.4%],  F:4.6%,M:9.5%,n:1013 | 42,7008 | 12,3738 | 1,3822 |
| Nextdenovo | 234.833 | 54 | 234.833 | 54 | 50.498.382 | 255 | C:59.9%[S:58.9%,D:1.0%],  F:6.5%,M:33.6%,n:1013 | 31,3455 | 58,0947 | 1,8161 |
| Nextdenovo + hypo | 234.328 | 54 | 234.328 | 54 | 50.435.433 | 255 | C:64.5%[S:63.1%,D:1.4%],  F:4.6%,M:30.9%,n:1013 | 41,7030 | 58,5576 | 1,7594 |
| Nextdenovo + hypo + purged | 235.030 | 53 | 235.030 | 53 | 49.913.310 | 253 | C:64.4%[S:63.4%,D:1.0%],  F:4.7%,M:30.9%,n:1013 | 42,5336 | 58,0388 | 1,1178 |
| Nextdenovo + hypo + purged +  10X scaffolding | 235.030 | 53 | 235.030 | 53 | 49.913.310 | 261 | C:64.4%[S:63.4%,D:1.0%],  F:4.7%,M:30.9%,n:1013 | 42,5348 | 58,0388 | 1,1177 |

**Merqury kmer spectra plots.**


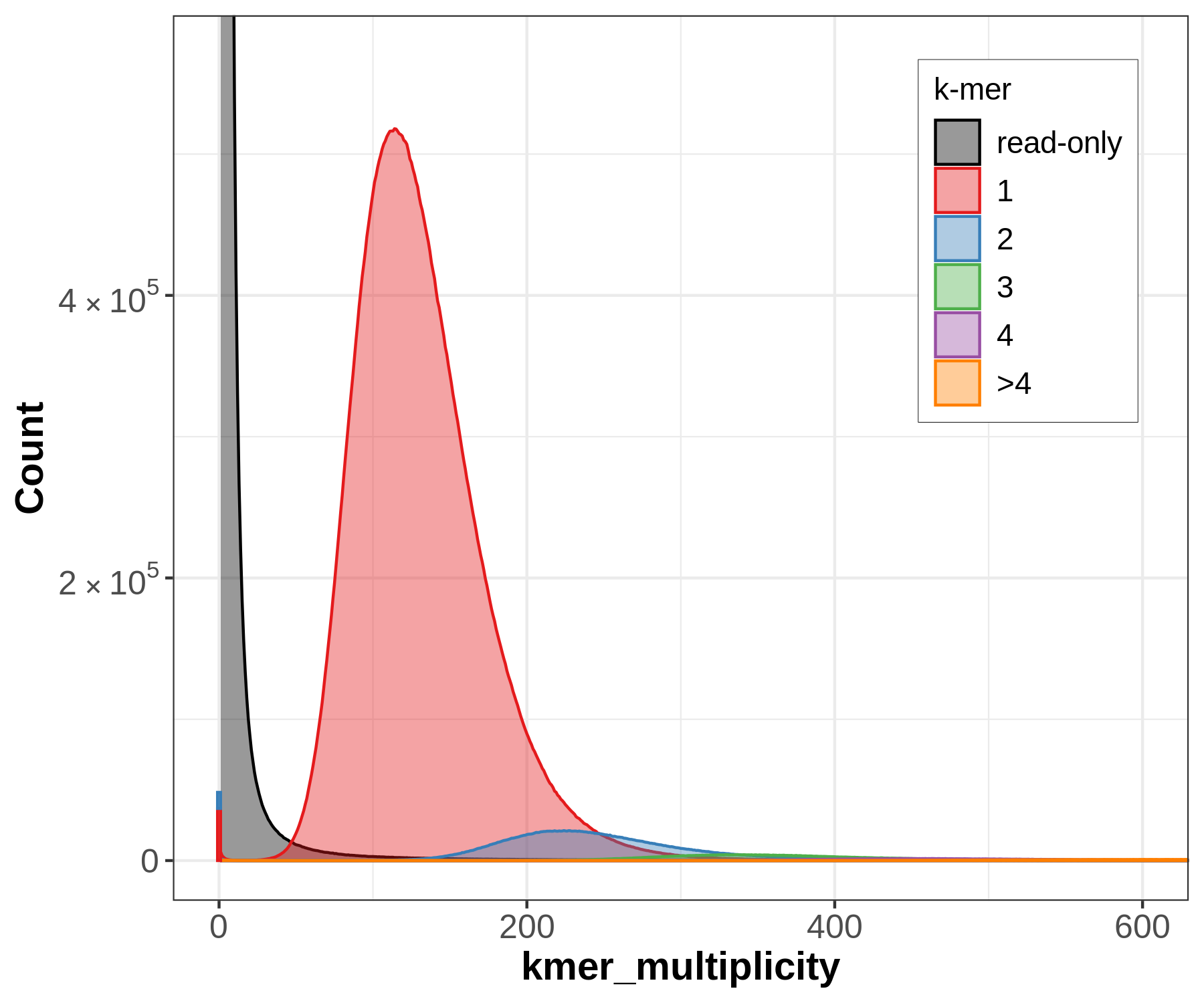


**Figure 3. qqPsoOvis1.1 Merqury K-mer spectra plot.**

**
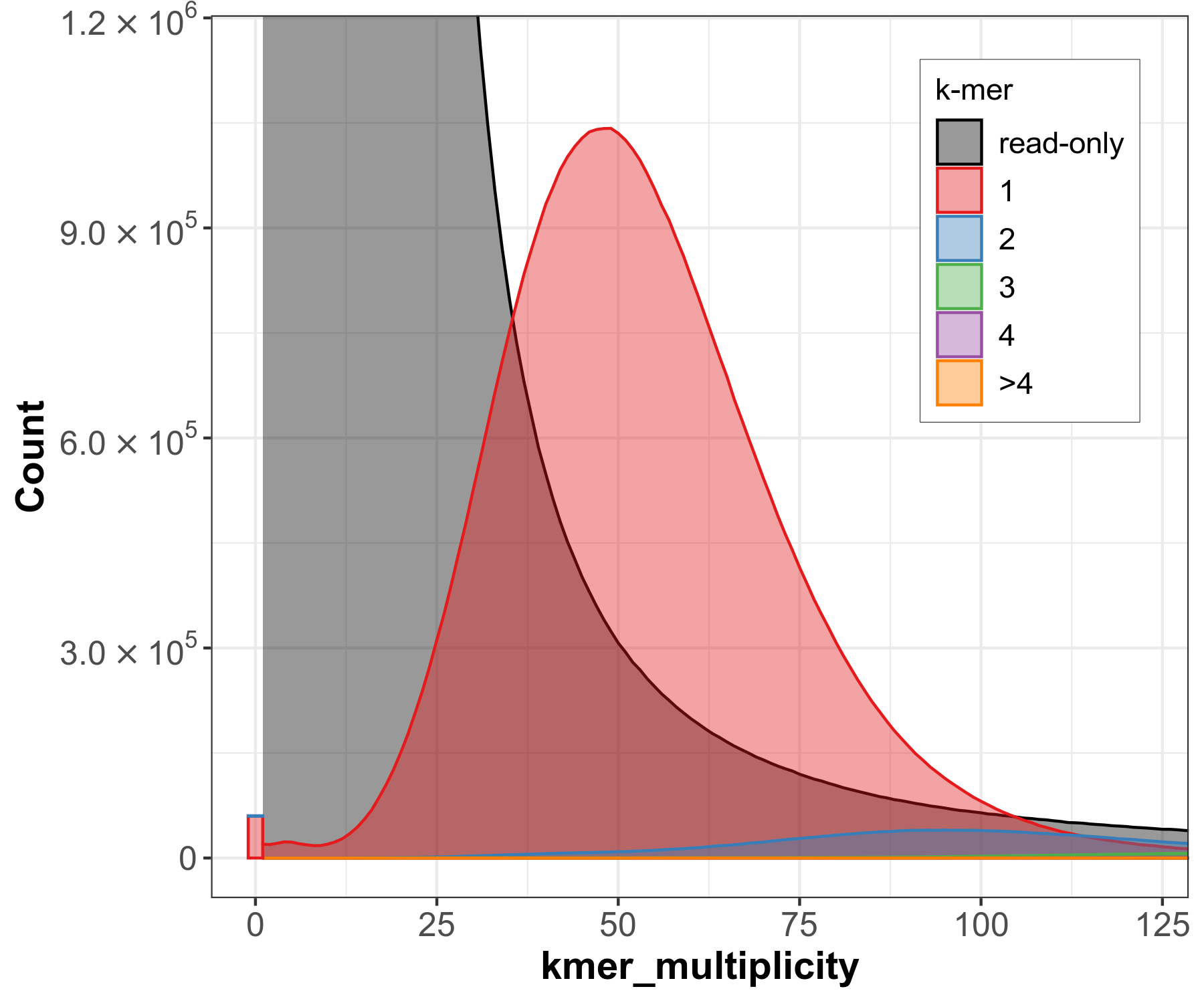
**

**Figure 4. qqPsoOvis2.1 Merqury K-mer spectra plot.**

**Assessing consistency of the sheep- and cattle-derived assemblies**

We aligned both genomes with DGENIES (15) (<https://dgenies.toulouse.inra.fr/>) to visualise consistency between assemblies and validate reference-based scaffolding using RagTag. The resulting plot (Figure 5) confirms that the chromosomal sections of both genomes are highly colinear. We just see a few repetitive sequences at the end that do not fully align.


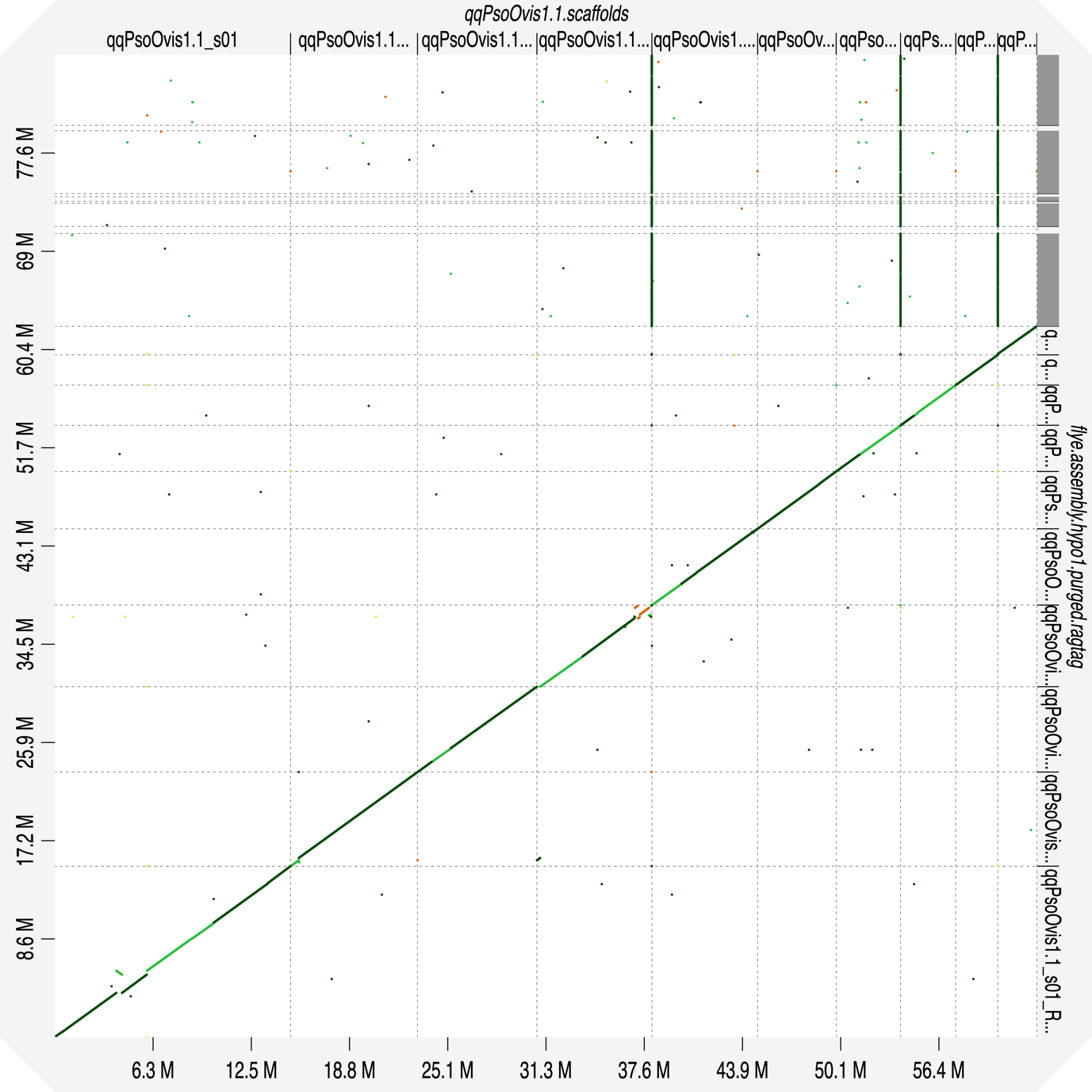


**Figure 5.** DGENIES alignment of sheep- and cattle-derived *P. ovis* assemblies.

## Acknowledgements

Institutional support to CNAG was from the Spanish Government, Ministry of Science, Innovation and Universities and Generalitat de Catalunya through the Departament de Recerca i Universitats and Departament de Salut.

## References

[1.    Marçais G, Kingsford C. A fast, lock-free approach for efficient parallel counting of occurrences of k-mers. Bioinformatics. 2011 Mar 15;27(6):764–70.](https://sciwheel.com/work/bibliography/1009601)

[2.    Ranallo-Benavidez TR, Jaron KS, Schatz MC. GenomeScope 2.0 and Smudgeplot for reference-free profiling of polyploid genomes. Nat Commun. 2020 Mar 18;11(1):1432.](https://sciwheel.com/work/bibliography/10208900)

[3.    Kolmogorov M, Yuan J, Lin Y, Pevzner PA. Assembly of long, error-prone reads using repeat graphs. Nat Biotechnol. 2019 May;37(5):540–6.](https://sciwheel.com/work/bibliography/6744810)

[4.    Kundu R, Casey J, Sung W-K. Hypo: super fast & accurate polisher for long read genome assemblies. BioRxiv. 2019 Dec 20;](https://sciwheel.com/work/bibliography/9175099)

[5.    Challis R, Richards E, Rajan J, Cochrane G, Blaxter M. BlobToolKit - Interactive Quality Assessment of Genome Assemblies. G3 (Bethesda). 2020 Apr 9;10(4):1361–74.](https://sciwheel.com/work/bibliography/8266351)

[6.    Jackman SD, Coombe L, Chu J, Warren RL, Vandervalk BP, Yeo S, et al. Tigmint: correcting assembly errors using linked reads from large molecules. BMC Bioinformatics. 2018 Oct 26;19(1):393.](https://sciwheel.com/work/bibliography/5956327)

[7.    Coombe L, Zhang J, Vandervalk BP, Chu J, Jackman SD, Birol I, et al. ARKS: chromosome-scale scaffolding of human genome drafts with linked read kmers. BMC Bioinformatics. 2018 Jun 20;19(1):234.](https://sciwheel.com/work/bibliography/8893228)

[8.    Warren RL, Yang C, Vandervalk BP, Behsaz B, Lagman A, Jones SJM, et al. LINKS: Scalable, alignment-free scaffolding of draft genomes with long reads. Gigascience. 2015 Aug 4;4:35.](https://sciwheel.com/work/bibliography/722355)

[9.    Gomez-Garrido J. CLAWS (CNAG’s long-read assembly workflow for Snakemake). WorkflowHub. 2023;](https://sciwheel.com/work/bibliography/16555987)

[10.   Hu J, Wang Z, Sun Z, Hu B, Ayoola AO, Liang F, et al. An efficient error correction and accurate assembly tool for noisy long reads. BioRxiv. 2023 Mar 12;](https://sciwheel.com/work/bibliography/15387919)

[11.   Guan D, McCarthy SA, Wood J, Howe K, Wang Y, Durbin R. Identifying and removing haplotypic duplication in primary genome assemblies. Bioinformatics. 2020 May 1;36(9):2896–8.](https://sciwheel.com/work/bibliography/8159585)

[12.   Alonge M, Lebeigle L, Kirsche M, Jenike K, Ou S, Aganezov S, et al. Automated assembly scaffolding using RagTag elevates a new tomato system for high-throughput genome editing. Genome Biol. 2022 Dec 15;23(1):258.](https://sciwheel.com/work/bibliography/14687793)

[13.   Manni M, Berkeley MR, Seppey M, Simão FA, Zdobnov EM. BUSCO Update: Novel and Streamlined Workflows along with Broader and Deeper Phylogenetic Coverage for Scoring of Eukaryotic, Prokaryotic, and Viral Genomes. Mol Biol Evol. 2021 Sep 27;38(10):4647–54.](https://sciwheel.com/work/bibliography/11841494)

[14.   Rhie A, Walenz BP, Koren S, Phillippy AM. Merqury: reference-free quality, completeness, and phasing assessment for genome assemblies. Genome Biol. 2020 Sep 14;21(1):245.](https://sciwheel.com/work/bibliography/9829443)

15. Cabanettes F, Klopp C. (2018) D-GENIES: dot plot large genomes in an interactive, efficient and simple way. PeerJ 6:e4958 <https://doi.org/10.7717/peerj.4958>.

**Supplementary Methods: Genome annotation report for** PsoOvis1B

Jessica Gomez-Garrido^1,2^, Tyler Alioto^1,2^

1. Centro Nacional de Análisis Genómico (CNAG), Baldiri Reixac 4, 08028 Barcelona, Spain.

2. Universitat de Barcelona (UB), Barcelona, Spain.

**Methods**

A library with repeats present in the qqPsoOvis1.1 genome assembly was built with RepeatModeler v1.0.11. After excluding those repeats that were part of repetitive protein families (performing a BLAST [(1)](https://sciwheel.com/work/citation?ids=215&pre=&suf=&sa=0&dbf=0) search against Uniprot) from the resulting library, RepeatMasker v4-1-2 ([http://www.repeatmasker.org)](about:blank) was run in order to annotate the repeats in the genome assembly.

The gene annotation of the *Psoroptes Ovis* genome assembly was obtained by combining transcript alignments, protein alignments and *ab initio*gene predictions.

Firstly, RNAseq reads downloaded from NCBI with the project accession number PRJNA521406 were aligned to the genome with STAR [(2)](https://sciwheel.com/work/citation?ids=49324&pre=&suf=&sa=0&dbf=0) v-2.7.2a. Transcript models were subsequently generated using Stringtie [(3)](https://sciwheel.com/work/citation?ids=111805&pre=&suf=&sa=0&dbf=0) v2.1.4 on each BAM file and then all the models produced were combined using TACO [(4)](https://sciwheel.com/work/citation?ids=2906083&pre=&suf=&sa=0&dbf=0) v0.6.3. High-quality junctions to be used during the annotation process were obtained by running Portcullis [(5)](https://sciwheel.com/work/citation?ids=6290211&pre=&suf=&sa=0&dbf=0) v1.2.0 after mapping with STAR. Finally, PASA assemblies were produced with PASA [(6)](https://sciwheel.com/work/citation?ids=1009281&pre=&suf=&sa=0&dbf=0) v2.4.1 by adding also the 1943 *Psoroptes ovis* ESTs downloaded from NCBI (last access January 2022). The *TransDecoder* program, which is part of the PASA package, was run on the PASA assemblies to detect coding regions in the transcripts.

Secondly, the complete *Tetranichus urticae*, *Sarcoptes scabiei*, *Euroglyphus maynei*, *Dermatophagoides pteronyssinus* and *Varroa destructor* proteomes were downloaded from Uniprot in January 2022 and aligned to the genome using Spaln [(7)](https://sciwheel.com/work/citation?ids=1397455&pre=&suf=&sa=0&dbf=0) v2.4.03. Alignments with very long introns (longer than 10 kb) were filtered out to prevent the annotation of nested genes made of tandemly repeated gene families. *Ab initio* gene predictions were performed on the repeat masked qqPsoOvis1.1 assembly with three different programs: GeneID [(8)](https://sciwheel.com/work/citation?ids=5909083&pre=&suf=&sa=0&dbf=0) v1.4, Augustus [(9)](https://sciwheel.com/work/citation?ids=964441&pre=&suf=&sa=0&dbf=0) v3.3.4 and Genemark-ES [(10)](https://sciwheel.com/work/citation?ids=1341464&pre=&suf=&sa=0&dbf=0) v2.3e with and without incorporating evidence from the RNAseq data. The gene predictors were run with trained parameters for honeybee, except Genemark that runs on a self-trained manner. Finally, all the data was combined into consensus CDS models using EvidenceModeler-1.1.1 (EVM) [(6)](https://sciwheel.com/work/citation?ids=1009281&pre=&suf=&sa=0&dbf=0). Additionally, UTRs and alternative splicing forms were annotated through two rounds of PASA annotation updates.  Functional annotation was performed on the annotated proteins with Blast2go [(11)](https://sciwheel.com/work/citation?ids=801833&pre=&suf=&sa=0&dbf=0). First, a Diamond Blastp [(12)](https://sciwheel.com/work/citation?ids=429288&pre=&suf=&sa=0&dbf=0) search was made against the nr database (last accessed January 2022). Furthermore, Interproscan [(13)](https://sciwheel.com/work/citation?ids=801564&pre=&suf=&sa=0&dbf=0) was run to detect protein domains on the annotated proteins. All these data were combined by Blast2go which produced the final functional annotation results.

The annotation of ncRNAs was produced by running the following steps. First, the program cmsearch [(14)](https://sciwheel.com/work/citation?ids=5923909&pre=&suf=&sa=0&dbf=0) v1.1 that comes with Infernal [(15)](https://sciwheel.com/work/citation?ids=2734726&pre=&suf=&sa=0&dbf=0) was run against the RFAM database of RNA families [(15)](https://sciwheel.com/work/citation?ids=2734726&pre=&suf=&sa=0&dbf=0) v12.0. Also, tRNAscan-SE [(16)](https://sciwheel.com/work/citation?ids=7862863&pre=&suf=&sa=0&dbf=0) v2.08 was run in order to detect the transfer RNA genes present in the genome assembly. To detect the lncRNAs we selected those Pasa-assemblies that had not been included into the annotation of protein-coding genes in order to get all those expressed genes that were not translated into a protein. Finally, those Pasa-assemblies without protein-coding gene annotation that were longer than 200bp and whose length was not covered at least in an 80% by a small ncRNA were incorporated into the ncRNA annotation as lncRNAs. The resulting transcripts were clustered into genes using shared splice sites or significant sequence overlap as criteria for designation as the same gene.

**Results**

In total, we have annotated 10,516 nuclear protein-coding genes, that produce 14,782 transcripts (1.4 transcripts per gene) and encode for 13,361 unique protein products. We have been able to assign functional labels to 61.62% of the annotated proteins. The annotated transcripts contain 4.27 exons on average, with 87% of them being multi-exonic (Table Annotation1). In addition, 10,565 non-coding transcripts have been annotated, of which 1,655 and 8,910 are long and short non-coding RNA genes, respectively.

**Table Annotation1: Genome annotation statistics**

|  | PsoOvis1B |
| --- | --- |
| Number of protein-coding genes | 10,516 |
| Median gene length (bp) | 1,903 |
| Number of transcripts | 14,782 |
| Number of exons | 46,576 |
| Number of coding exons | 42,137 |
| Median UTR length (bp) | 404 |
| Median intron length (bp) | 75 |
| Exons/transcript | 4.27 |
| Transcripts/gene | 1.4 |
| Multi-exonic transcripts | 0.87 |
| Gene density (gene/Mb) | 167.8 |

**References**

[1.    Altschul SF, Gish W, Miller W, Myers EW, Lipman DJ. Basic local alignment search tool. J Mol Biol. 1990 Oct 5;215(3):403–10.](https://sciwheel.com/work/bibliography/215)

[2.    Dobin A, Davis CA, Schlesinger F, Drenkow J, Zaleski C, Jha S, et al. STAR: ultrafast universal RNA-seq aligner. Bioinformatics. 2013 Jan 1;29(1):15–21.](https://sciwheel.com/work/bibliography/49324)

[3.    Pertea M, Pertea GM, Antonescu CM, Chang T-C, Mendell JT, Salzberg SL. StringTie enables improved reconstruction of a transcriptome from RNA-seq reads. Nat Biotechnol. 2015 Mar;33(3):290–5.](https://sciwheel.com/work/bibliography/111805)

[4.    Niknafs YS, Pandian B, Iyer HK, Chinnaiyan AM, Iyer MK. TACO produces robust multisample transcriptome assemblies from RNA-seq. Nat Methods. 2017 Jan;14(1):68–70.](https://sciwheel.com/work/bibliography/2906083)

[5.    Mapleson D, Venturini L, Kaithakottil G, Swarbreck D. Efficient and accurate detection of splice junctions from RNA-seq with Portcullis. Gigascience. 2018 Dec 1;7(12).](https://sciwheel.com/work/bibliography/6290211)

[6.    Haas BJ, Salzberg SL, Zhu W, Pertea M, Allen JE, Orvis J, et al. Automated eukaryotic gene structure annotation using EVidenceModeler and the Program to Assemble Spliced Alignments. Genome Biol. 2008 Jan 11;9(1):R7.](https://sciwheel.com/work/bibliography/1009281)

[7.    Iwata H, Gotoh O. Benchmarking spliced alignment programs including Spaln2, an extended version of Spaln that incorporates additional species-specific features. Nucleic Acids Res. 2012 Nov 1;40(20):e161.](https://sciwheel.com/work/bibliography/1397455)

[8.    Alioto T, Blanco E, Parra G, Guigó R. Using geneid to Identify Genes. Curr Protoc Bioinformatics. 2018 Dec;64(1):e56.](https://sciwheel.com/work/bibliography/5909083)

[9.    Stanke M, Schöffmann O, Morgenstern B, Waack S. Gene prediction in eukaryotes with a generalized hidden Markov model that uses hints from external sources. BMC Bioinformatics. 2006 Feb 9;7:62.](https://sciwheel.com/work/bibliography/964441)

[10.   Lomsadze A, Burns PD, Borodovsky M. Integration of mapped RNA-Seq reads into automatic training of eukaryotic gene finding algorithm. Nucleic Acids Res. 2014 Sep;42(15):e119.](https://sciwheel.com/work/bibliography/1341464)

[11.   Conesa A, Götz S, García-Gómez JM, Terol J, Talón M, Robles M. Blast2GO: a universal tool for annotation, visualization and analysis in functional genomics research. Bioinformatics. 2005 Sep 15;21(18):3674–6.](https://sciwheel.com/work/bibliography/801833)

[12.   Buchfink B, Xie C, Huson DH. Fast and sensitive protein alignment using DIAMOND. Nat Methods. 2015 Jan;12(1):59–60.](https://sciwheel.com/work/bibliography/429288)

[13.   Jones P, Binns D, Chang H-Y, Fraser M, Li W, McAnulla C, et al. InterProScan 5: genome-scale protein function classification. Bioinformatics. 2014 May 1;30(9):1236–40.](https://sciwheel.com/work/bibliography/801564)

[14.   Cui X, Lu Z, Wang S, Jing-Yan Wang J, Gao X. CMsearch: simultaneous exploration of protein sequence space and structure space improves not only protein homology detection but also protein structure prediction. Bioinformatics. 2016 Jun 15;32(12):i332–40.](https://sciwheel.com/work/bibliography/5923909)

[15.   Nawrocki EP, Eddy SR. Infernal 1.1: 100-fold faster RNA homology searches. Bioinformatics. 2013 Nov 15;29(22):2933–5.](https://sciwheel.com/work/bibliography/2734726)

[16.   Chan PP, Lowe TM. tRNAscan-SE: Searching for tRNA Genes in Genomic Sequences. Methods Mol Biol. 2019;1962:1–14.](https://sciwheel.com/work/bibliography/7862863)
