# Supplementary figures and images for: Over-expression and increased copy numbers of a cytochrome P450 and two UDP-glucuronosyltransferase genes in macrocyclic lactone resistant Psoroptes ovis of cattle
Source: PLoS Pathog. 2025 Jul 29;21(7):e1012963. doi: 10.1371/journal.ppat.1012963 (PMC12364353; doi:10.1371/journal.ppat.1012963)

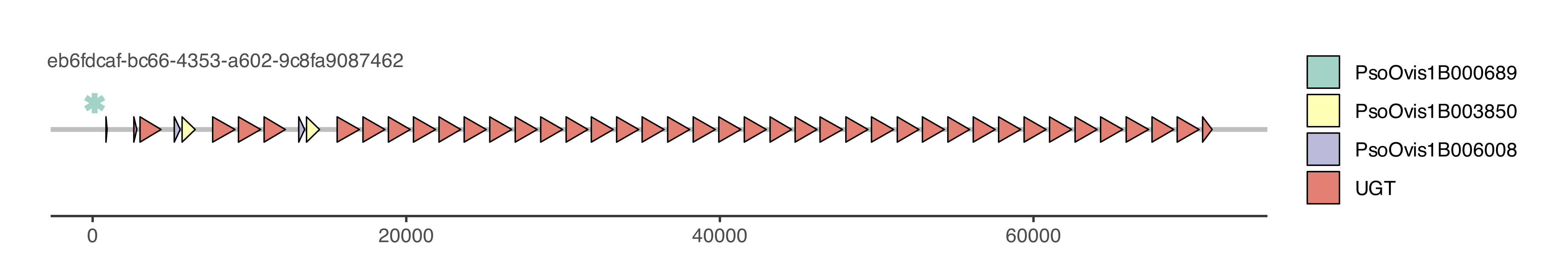

Supplement: S2 Fig — A coloured asterisk is given for PsoOvis1B00689 as it is too short to colour the gene arrow. (TIFF) [file ppat.1012963.s017.tiff]

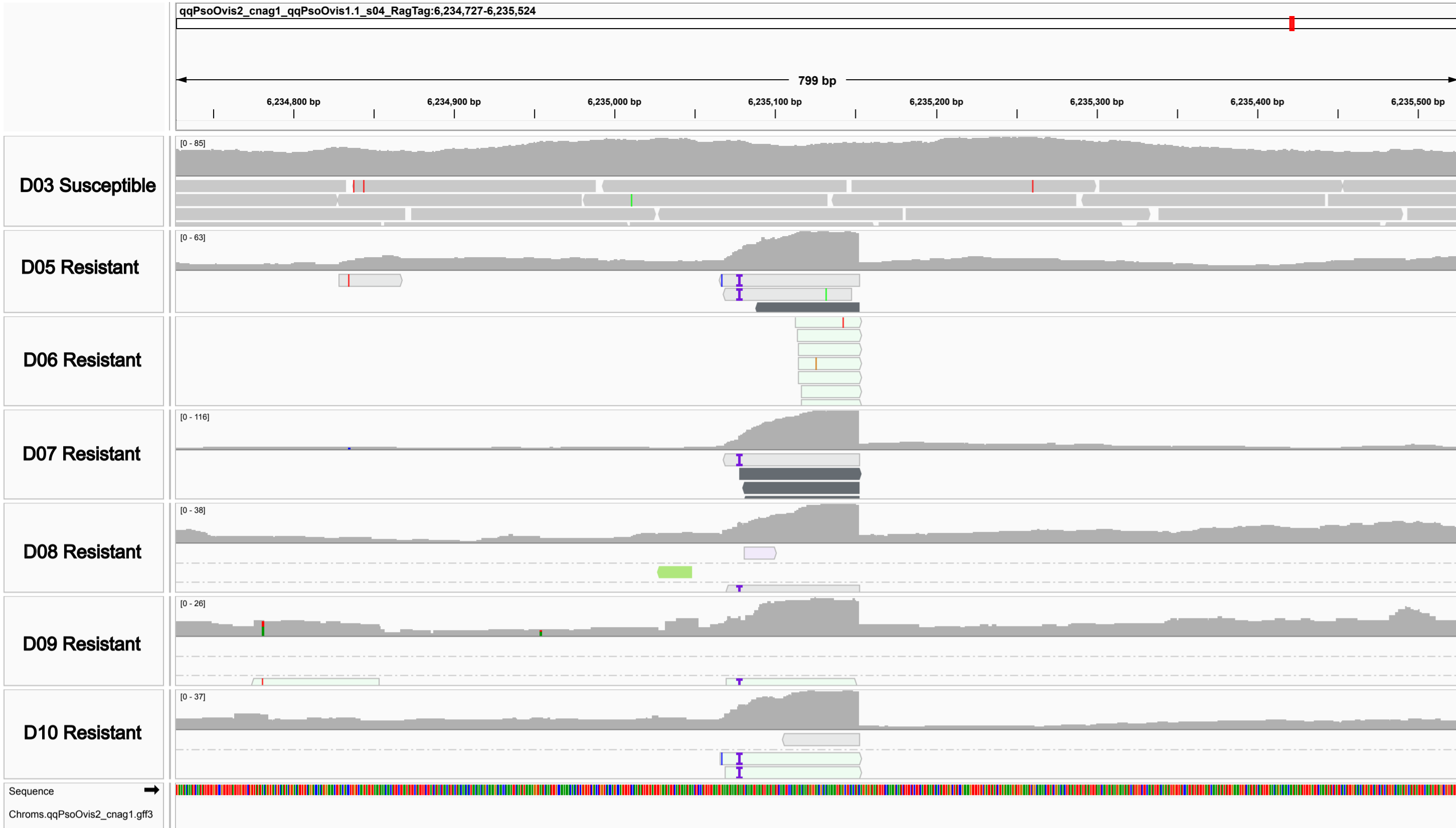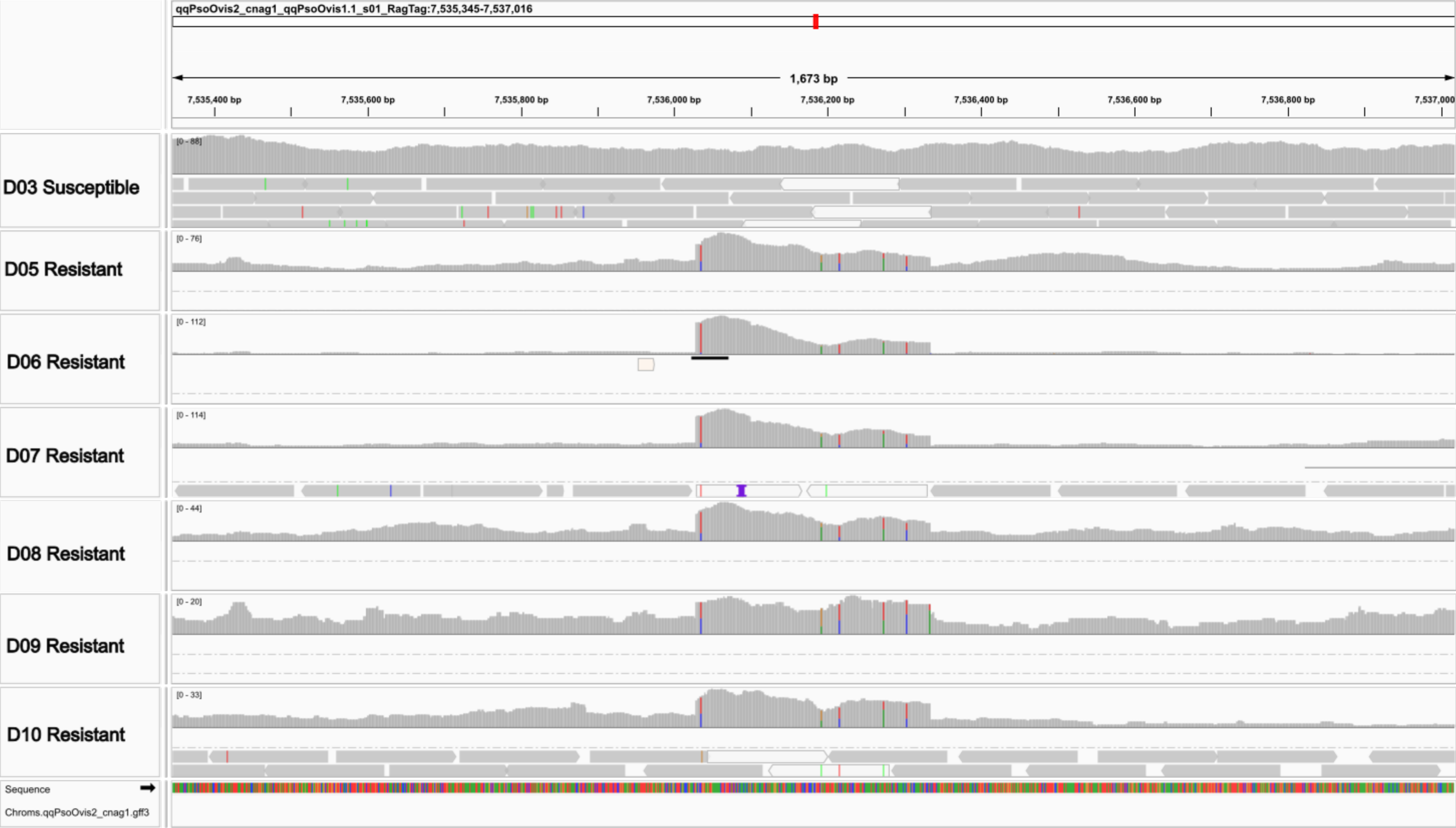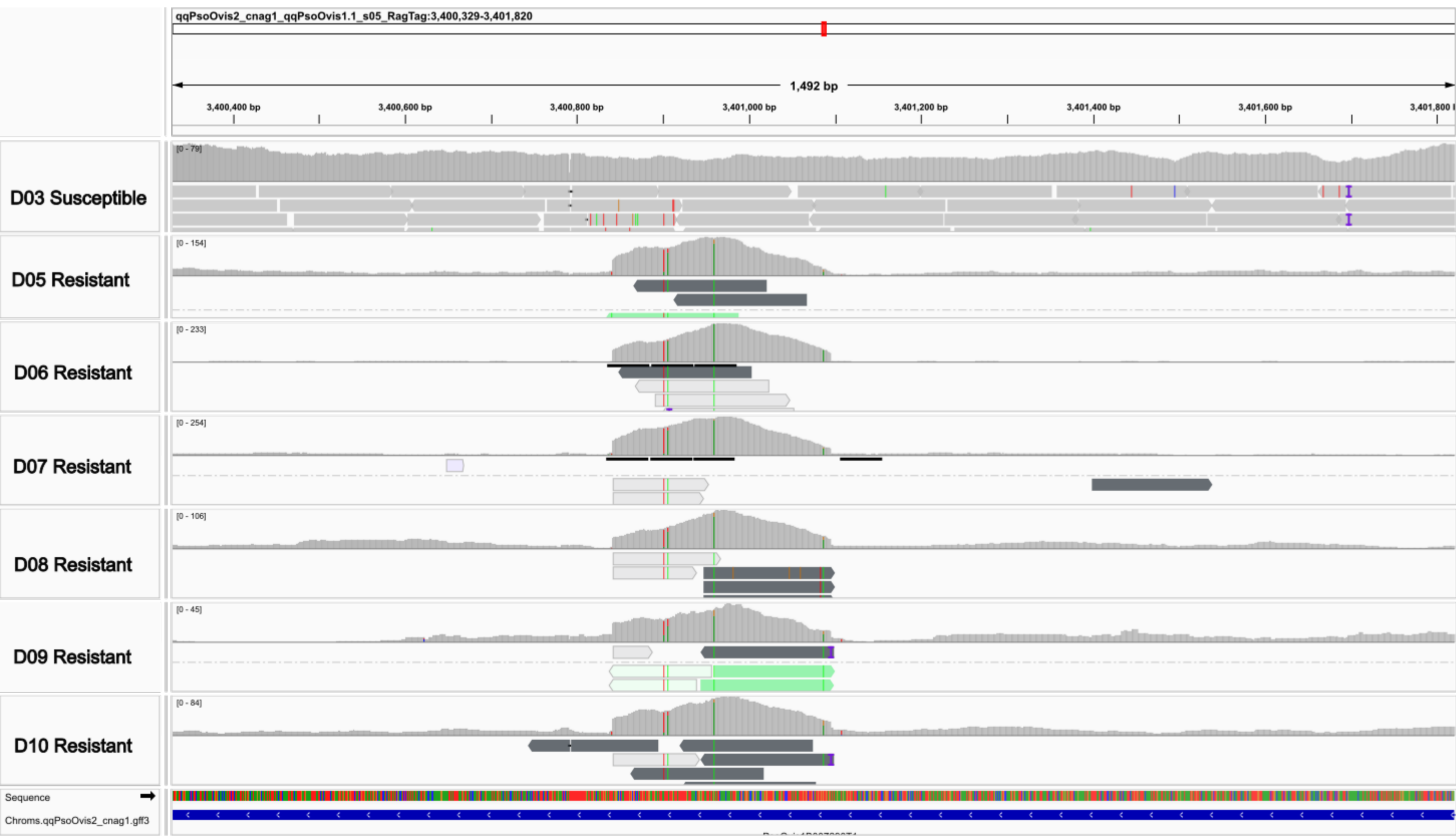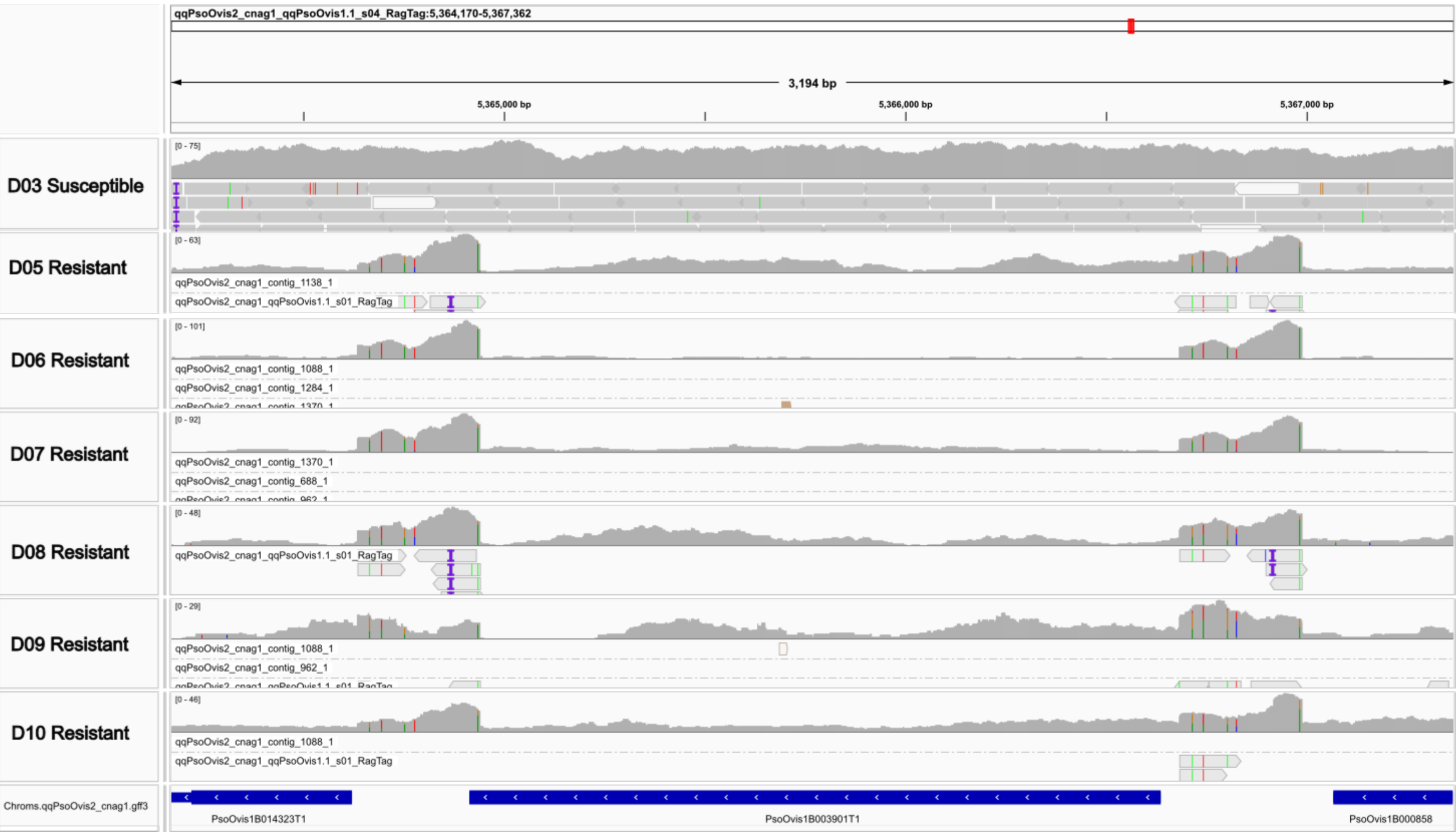

Supplement: S3 Fig — Five possible positions at four locations in the genome are shown by excessive read coverage with discordant read-mapping of pairs with the PsoOvis1B011549 locus. (PDF) [file ppat.1012963.s018.pdf]
